# Effects of Ciprofloxacin on the Production and Composition of Cellular Microcystins in *Microcystis aeruginosa*

**DOI:** 10.3390/toxics12100759

**Published:** 2024-10-19

**Authors:** Liang Wan, Rong Huang, Yan Zhou, Jiahao Guo, Yiying Jiao, Jian Gao

**Affiliations:** 1 School of Civil Engineering, Architecture and Environment, Hubei University of Technology, Wuhan 430068, China; 2 Key Laboratory of Intelligent Health Perception and Ecological Restoration of Rivers and Lakes, Ministry of Education, Hubei University of Technology, Wuhan 430068, China; 3 Innovation Demonstration Base of Ecological Environment Geotechnical and Ecological Restoration of Rivers and Lakes, Hubei University of Technology, Wuhan 430068, China

**Keywords:** fluoroquinolones, *Microcystis aeruginosa*, microcystins, production and composition

## Abstract

Antibiotics can affect the photosynthetic system of *Microcystis*, potentially altering the balance of carbon and nitrogen, which may influence the synthesis of different microcystin (MC) congeners. However, the regulatory mechanisms by which antibiotics affect the synthesis of various MC congeners in *Microcystis* remain unknown. In this study, the effects of ciprofloxacin (CIP) on the growth, carbon and nitrogen balance, amino acid composition, *mcyB* gene expression, and production of different MC congeners were investigated in two toxin-producing strains of *Microcystis aeruginosa*. The results show that CIP exposure significantly inhibited the growth of both strains, achieving an inhibition rate of 71.75% in FACHB-315 and 41.13% in FACHB-915 at 8 μg/L CIP by the end of the cultivation. The intracellular C:N ratio in FACHB-315 increased by 51.47%, while no significant change was observed in FACHB-915. The levels of leucine, tyrosine, and arginine, as identified and quantified by UPLC-MS/MS, were significantly altered at higher CIP concentrations, leading to a reduction in leucine percentage and a notable increase in tyrosine in both strains, which contributed to a reduction in MC-LR proportion and an increase in MC-RR and MC-YR proportion. Additionally, the expression of the *mcyB* gene was upregulated by as much as 5.57 times, indicating that antibiotic stress could enhance MC synthesis at the genetic level, contributing to the increased toxicity of cyanobacteria. These findings emphasize the significant role of CIP in the biochemical processes of *M. aeruginosa*, particularly in MC synthesis and composition, providing valuable insights into the ecological risks posed by antibiotics and harmful cyanobacteria.

## 1. Introduction

The extensive use of antibiotics in healthcare, agriculture, and aquaculture has raised significant concerns about antibiotic pollution in the environment. Originally designed to combat bacterial infections, these drugs now cause environmental problems and pose considerable threats to ecosystems and human health due to their wide application and subsequent release into natural environments [1]. Antibiotics are commonly categorized based on their structure, with major groups such as fluoroquinolones, tetracyclines, macrolides, etc. Fluoroquinolones are particularly notable for their low cost, broad-spectrum antimicrobial activity, and high efficacy [2]. They are extensively used for the prevention and treatment of diseases in humans and livestock [3], and their amount of usage ranks among the highest in both clinical and agricultural sectors in China [4]. Common fluoroquinolones include second-generation ciprofloxacin (CIP), norfloxacin (NOR), enrofloxacin (ENR), third-generation levofloxacin (LEV), and fourth-generation moxifloxacin (MOX) [5]. Fluoroquinolones are not easily absorbed or metabolized by humans and animals, resulting in a large proportion being excreted in their original form after drug consumption [6]. Due to their stable chemical structures, they are resistant to hydrolysis and degrade slowly in aquatic environments [7]. Furthermore, wastewater treatment systems have low removal efficiency for fluoroquinolones, resulting in their persistent residues in aquatic environments, which pose great threats to human health and aquatic ecosystems [8].

Fluoroquinolone antibiotics are frequently detected in rivers and lakes across China, with concentrations typically ranging from ng/L to μg/L. For instance, in the Liao River, the concentrations of ofloxacin (OFL) and NOR were detected to be 632.53 ng/L and 256.03 ng/L, respectively [9]. In the Hai River, the concentrations of NOR and CIP reached as high as 5770 ng/L and 1290 ng/L, respectively [10]. In Dianchi Lake, the concentration of OFL was measured to be 713.6 ng/L [11]. The frequent detection of high concentrations of fluoroquinolones identifies them as antibiotics that pose the highest ecological risks in China [12]. Although antibiotic misuse has been somewhat controlled in China in recent years, antibiotic pollution remains severe, and the associated ecological risks and environmental impacts of antibiotic residues continue to pose significant challenges. Fluoroquinolone antibiotics in aquatic environments are known to exert toxic effects on nontarget aquatic organisms, such as cyanobacteria, green algae, duckweed, daphnia, and fish. Among aquatic organisms, cyanobacteria are particularly sensitive to fluoroquinolones. These antibiotics can impact the growth of cyanobacteria at environmentally relevant concentrations [13,14,15].

The outbreak of *Microcystis* blooms has raised further concerns due to their production of toxic metabolites, which severely compromise the safety of drinking water and likely affect aquatic ecosystems. Microcystins (MCs), secondary metabolites produced by *Microcystis*, are potent hepatotoxins with high carcinogenic potential. These toxins can bioaccumulate and spread within food webs in aquatic ecosystems, posing substantial risks to both public health and ecosystem health [16]. MCs are cyclic heptapeptides, and their structural variants arise from differences in amino acid composition. The most common and widespread MC variants are MC-LR, MC-RR, and MC-YR, though many other MC congeners, such as MC-LA, have also been reported increasingly [17]. For instance, the primary MC congener in some lakes in Canada and the United States is MC-LA [18]. The toxicity of different MC congeners varies significantly. For example, the LD_50_ of MC-RR for mice is 600 μg/L, whereas for MC-LR, it is only 50 μg/L [19], suggesting MC-LR is notably more toxic than MC-RR to mice. Environmental factors, such as elevated CO_2_ concentrations, favor the synthesis of high C ratio isomers, leading to increased toxin potency [19]. Low concentrations of lanthanum can increase both the total concentration of MCs and the proportion of highly toxic MC congeners, while high concentrations of lanthanum have the opposite effect [20]. Nutrient limitations (N and P) and fluoroquinolone antibiotics can both elevate the total intracellular and extracellular microcystin concentrations [21,22,23]. Fluoroquinolone antibiotics can affect the photosynthetic system of *Microcystis*, potentially altering the carbon and nitrogen balance under varying nutrient conditions, which may, in turn, affect the synthesis of different MC congeners. However, limited research has been conducted on the mechanisms by which antibiotics regulate the synthesis of different MC congeners in *Microcystis*. This knowledge gap restricts our ability to assess the pollution levels and toxicity of various MC congeners during *Microcystis* blooms. Therefore, it is essential to conduct research on how antibiotics influence MC synthesis, particularly focusing on their role in the production of different MC congeners.

In this study, the effects of environmental concentrations of CIP on the production and composition of cellular MCs in *M. aeruginosa* were investigated. Two strains of *M. aeruginosa* (FACHB-915 and FACHB-315) were selected for investigation, and both strains are known to produce the toxins MC-LR, MC-RR, and MC-YR. The influence of CIP on algal growth, changes in cellular C:N ratios, amino acid synthesis, gene expression related to MC production, and the levels of MCs within the cells were assessed. This study aims to understand how fluoroquinolones affect the physiological and genetic processes related to MC production in these toxin-producing strains of *M. aeruginosa*, thereby contributing to a better understanding of the ecological risks of antibiotics on harmful cyanobacteria.

## 2. Materials and Methods

### 2.1. Microalgal Strains and Culture Condition

Two strains of *M. aeruginosa* (FACHB-915 and FACHB-315) were obtained from the Freshwater Algae Culture Collection, Institute of Hydrobiology, Chinese Academy of Sciences (Wuhan, China). Both strains are toxin-producing strains that could synthesize and release MC-LR, MC-RR, and MC-YR (where L, R, and Y represent leucine, arginine, and tyrosine, respectively), which are among the most common toxins produced by *M. aeruginosa* [24,25]. The strains were precultivated in sterilized BG11 medium under controlled conditions: 25 ± 1 °C, with a light intensity of 3000 lx and a 12 h light/dark cycle.

### 2.2. Antibiotic Treatment

CIP (≥98% purity) was purchased from Aladdin (Shanghai, China). A stock solution of 5 mg/L CIP was prepared in deionized water and stored at 4 °C. Prior to the experiment, the stock solution was filtered through a 0.22 μm membrane to ensure sterilization. CIP stock solution was added to the culture during the logarithmic growth phase of *M. aeruginosa*, achieving final CIP concentrations of 0, 1, 2, 4, and 8 μg/L. *M. aeruginosa* cultured without CIP (0 μg/L) served as the control group. The initial absorbance of the two *M. aeruginosa* strains was set at approximately 0.120, corresponding to an initial algal cell count of around 3.1 × 10^6^ cells/mL for FACHB-915 and 3.9 × 10^6^ cells/mL for FACHB-315. Cultures were maintained in Erlenmeyer flasks under the conditions described above for 18 days, with flasks shaken three times daily to prevent cell precipitation. Each treatment was conducted in triplicate in this study.

### 2.3. Analytical Procedures

#### 2.3.1. Determination of Algal Growth

The growth of *M. aeruginosa* was measured every two days at 680 nm using a UV/VIS spectrophotometer (Shimadzu, Kyoto, Japan). A standard regression equation was established between absorbance values and corresponding cell counts using a spectrophotometer and hemocytometer, respectively. The regression equations of FACHB-915 and FACHB-315 were established as follows and employed to compute cell density.

FACHB-315: y = 53.42x − 2.42 (R^2^ = 0.99)

FACHB-915: y = 51.35x − 3.13 (R^2^ = 0.99)

where y represents the cell density (10^6^ cells/mL) and x represents OD_680_, respectively.

#### 2.3.2. Determination of Intracellular Carbon (C) and Nitrogen (N) Content

After 18 days of cultivation, the *M. aeruginosa* cultures were centrifugated at 8000 rpm for 10 min at 4 °C. The cell pellets were washed with deionized water to remove any residual medium, freeze-dried, and stored at −70 °C for further analysis. The C content in algal cells was determined using the potassium dichromate–sulfuric acid oxidation method [26]. The N content of *M. aeruginosa* was determined by the alkaline potassium persulfate oxidation–UV spectrophotometric method [27].

#### 2.3.3. Determination of Amino Acid Content

Amino acid concentrations were determined according to the methods described by reference [28]. Freeze-dried algal powder was hydrolyzed with hydrochloric acid in a hydrolysis tube, followed by purging with nitrogen to displace the air before sealing. The mixture was then hydrolyzed in an oven at 115 ± 5 °C for 22 h. The cooled solution was then filtered, and a measured amount of filtrate was dried. The dried residue was redissolved in 0.1 M HCl for analysis.

The amino acid content was analyzed by the Ultimate 3000 UHPLC-Q Exactive system (Thermo Scientific, Waltham, MA, USA). The chromatographic column was Agilent ZORBAX SB-C18 (2.1 × 50 mm, 5 μm pore size) (Santa Clara, CA, USA), with a flow rate of 0.2 mL/min. The mobile phase consists of 0.1% formic acid in water (A) and 0.1% formic acid in acetonitrile (B). The gradient elution program was as follows: 0–2 min, 10% B; 2–2.1 min, 10% B to 90% B; 2.1–4 min, 90% B; 4–4.01 min, 90%B to 10% B; and 4.01–6 min, 10% B. The injection volume was 10 μL. The instrument was operated in positive ion mode with a capillary voltage of 3.5 kV. MS data were collected and analyzed using Xcalibur 3.0 software. The content of amino acid was quantitatively analyzed by an external standard method. The standard equations for amino acid concentration are as follows:

Leucine: y = 1.78 × 10^−7^ x − 226.97 (R^2^ = 0.95)

Arginine: y = 3.46 × 10^−7^ x − 1.09 (R^2^ = 0.99)

Tyrosine: y = 1.82 × 10^−7^ x + 9.15 (R^2^ = 0.99)

where x is the peak area and y is the amino acid content in ng, respectively.

#### 2.3.4. Determination of mcyB Genes Expression

The *mcyB* gene is crucial for the synthesis of MCs in *Microcystis*, as it is part of the *mcy* gene cluster responsible for producing MCs. The expression of *mcyB* genes was assessed by real-time PCR. A total of 15 mL of algal culture was centrifugated at 8000 rpm for 10 min at 4 °C, with the supernatant discarded. The algal pellet was resuspended in Trizol reagent, and the total RNA was extracted [29]. Real-time PCR was performed with 10 μL Master Mix and 0.2 μL forward primer and reverse primer, respectively. The primers of 16S rRNA and *mcyB* were designed according to Shao for real-time PCR [30]. The amplification reactions were performed under the following conditions: one cycle of denaturation at 95 °C for 3 min, followed by 40 cycles of 95 °C for 15 s, 59 °C for 30 s, 72 °C for 30 s. All of the samples were amplified in triplicate. *McyB* gene expression data from real-time PCR were evaluated using Ct value, with the 16S rRNA as the control [30].

#### 2.3.5. Determination of Intercellular MCs

The culture solution was centrifuged at 8000 rpm for 10 min at 4 °C. The cell pellets were resuspended with PBS to remove residual MCs and centrifuged again. The pellets were sonicated in an ice bath for cell disruption with 75% methanol. Then, the obtained solution was filtrated, and the supernatants were used for the determination of the intercellular MCs.

The content of the MCs was analyzed by the Ultimate 3000 UHPLC-Q Exactive system (Thermo Scientific, MA, USA). The mobile phase and the parameters of LC-MS\MS are the same as described previously. The gradient elution program is as follows: 0–1.5 min, 20% B; 1.5–3 min, 20% B to 95%B; 3–5 min, 95% B; 5–5.01 min, 95% B to 20% B; and 5.01–7 min, 20%B. The content of the MCs was quantitatively analyzed by external standard method, and the standard equations are as follows:

MC-LR: y = 4.55 × 10^−8^ x − 0.21 (R^2^ = 0.99)

MC-YR: y = 5.76 × 10^−8^ x + 3.18 × 10^−3^ (R^2^ = 0.99)

MC-RR: y = 2.28 × 10^−6^ x + 0.035 (R^2^ = 0.99)

where x is the peak area and y is the content of MCs in ng, respectively.

#### 2.3.6. Statistical Analyses

Experimental data were analyzed using IBM SPSS version 19.0 (IBM Corp., Armonk, NY, USA). Data are expressed as means ± S.D. One-way ANOVA through the Tukey–Kramer multiple comparison test was applied to evaluate significant differences between the control and treatment groups. Normality and homogeneity were verified using the Shapiro–Wilk test and Fligner–Killeen test, respectively. The significance differences were considered at *p* < 0.05.

## 3. Results and Discussion

### 3.1. Effects of CIP on Cellular Growth

**Figure 1:**
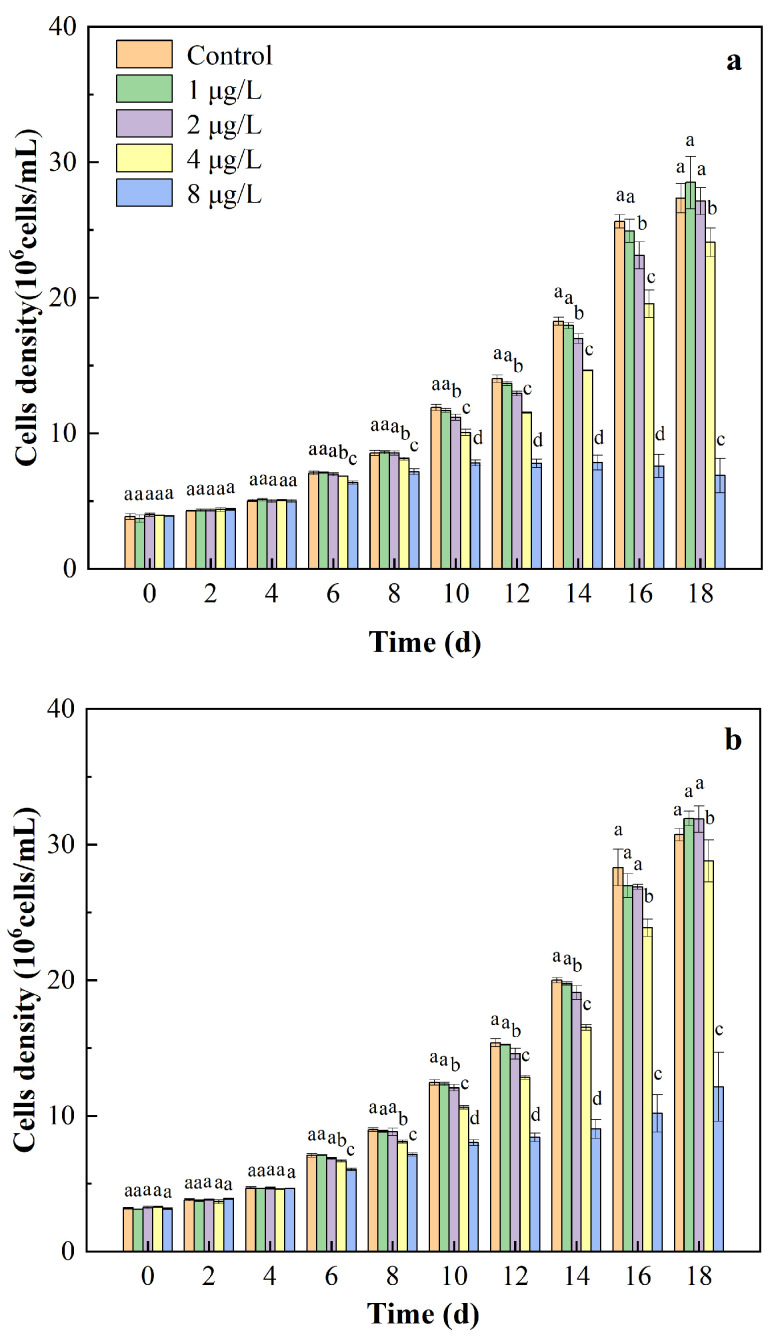
Effects of CIP concentrations on the growth of FACHB-315 (**a**) and FACHB-915 (**b**). Different letters indicate significant differences (*p* < 0.05) between the control and experimental groups.

The effects of different CIP concentrations on the growth of *M. aeruginosa* strains FACHB-315 and FACHB-915 are shown in Figure 1. During the initial 4 days of exposure, the growth of the two strains exposed to CIP did not show a significant difference compared with that in the control group. However, as the exposure time increased to 6 and 8 days, a notable decrease in cell growth was observed for both FACHB-315 and FACHB-915 at CIP concentrations above 4 μg/L. After 10 days of exposure, the cell growth of *M. aeruginosa* significantly decreased in all treatment groups except for the 1 μg/L CIP. No significant decreases in cell growth of both FACHB-315 and FACHB-915 were observed in the 1 μg/L CIP group for either strain throughout the 18 days of cultivation. By the end of the cultivation, the growth of FACHB-315 was inhibited by 7.69% and 71.75% at 4 and 8 μg/L CIP concentrations, respectively. Similarly, FACHB-915 was inhibited by 4.31% and 41.13% at the same concentrations. The high inhibition rates indicate that FACHB-315 is more sensitive to CIP than FACHB-915.

Antibiotics inhibit cyanobacterial growth primarily due to structural and functional similarities shared between cyanobacteria and bacteria. As prokaryotic organisms, cyanobacteria lack a nucleus and possess similar cell wall structures and metabolic pathways as bacteria. Antibiotics typically target specific structures or physiological processes in bacteria, such as cell wall synthesis, protein synthesis, or DNA replication. These similarities allow antibiotics to interfere with cyanobacterial cell functions, leading to growth inhibition. The inhibitory mechanisms of antibiotics on cyanobacteria can vary significantly, depending on the type and concentration of the antibiotics used. For example, sulfonamide antibiotics inhibit bacterial growth by competitively blocking the enzyme dihydropteroate synthetase, a disrupting folic acid synthesis essential for DNA and RNA production [31]. Sulfamethoxazole, a sulfonamide antibiotic, inhibited the growth of *M. aeruginosa* but stimulated esterase activity and photosynthesis at concentrations of 50 and 125 µg/L [32]. Tetracycline antibiotics, on the other hand, inhibit bacterial growth by binding to the 30S ribosomal subunit, thereby blocking the attachment of tRNA to the mRNA-ribosome complex and preventing protein synthesis [33]. Studies showed that three tetracycline antibiotics, oxytetracycline hydrochloride, tetracycline hydrochloride, and chlortetracycline hydrochloride, exhibited an insignificant effect on *M. aeruginosa* at concentrations below 100 μg/L [22]. Fluoroquinolones, such as CIP, inhibit bacterial growth by targeting DNA gyrase and topoisomerase IV, enzymes essential for DNA replication and transcription, thereby disrupting bacterial DNA synthesis [4]. In this study, it was observed that FACHB-315 exhibited greater sensitivity to CIP exposure compared with FACHB-915, highlighting strain-specific variations in response to CIP exposure. Previous studies have reported that another two strains of *M. aeruginosa* EAWAG 198 and *M. aeruginosa* LE3 were significantly inhibited by CIP at concentrations higher than 50 μg/L and 10 μg/L, respectively, after 96 h of exposure [34]. However, the 96 h EC_50_ values of CIP for *M. aeruginosa* FACHB-930 were reported to be 49.80 mg/L [35], which is thousands of times higher than the concentrations used in other studies. Moreover, the growth of *M. aeruginosa* NIES-843 was significantly stimulated by CIP concentrations ranging from 50 to 200 ng/L after 15 days of cultivation [36]. The great distinction of sensitivity among different strains of the same species suggests that strain-specific characteristics play a crucial role in determining the outcome of antibiotic exposures, highlighting the importance of selecting appropriate algal species when conducting toxicological assessments.

### 3.2. Effects of CIP on the Ratios of C:N

**Figure 2:**
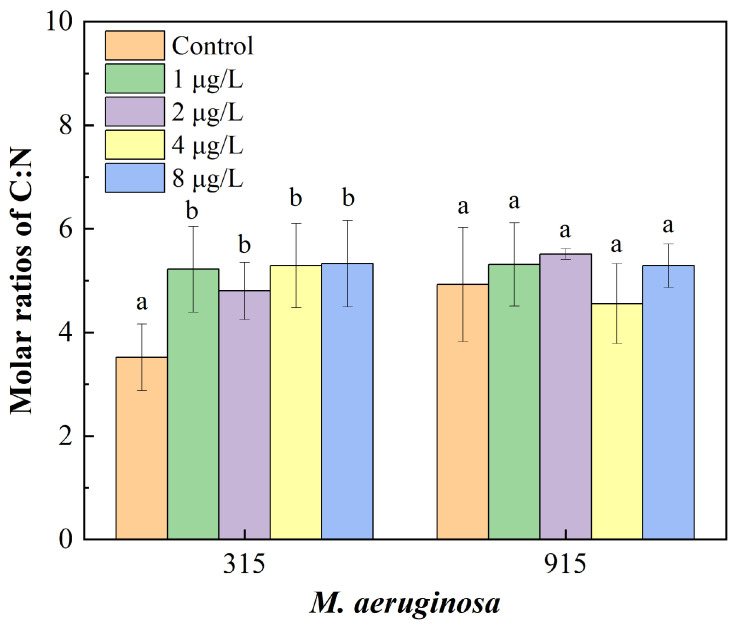
Effects of CIP concentrations on molar ratio of C:N in FACHB-315 and FACHB-915 after 18 days of cultivation. Different letters indicate significant differences (*p* < 0.05) between the control and experimental groups.

The effects of CIP on the molar ratio of C:N in *M. aeruginosa* are presented in Figure 2. The molar ratios of C:N in FACHB-315 increased significantly under CIP exposure. Compared with the control, the C:N ratio in FACHB-315 increased by 48.84%, 36.48%, 50.41%, and 51.47% when exposed to CIP concentrations at 1, 2, 4, and 8 μg/L, respectively. In contrast, the C:N ratio in FACHB-915 showed no significant changes under the same CIP concentrations.

C and N are essential elements of organisms for the synthesis of key organic compounds, including carbohydrates, fatty acids, amino acids, proteins, and nucleic acids [37]. They play a crucial role in performing the routine and fundamental cellular activities of algae and influence the growth, development, and response of algae to a wide array of stresses. The C:N ratio in algae is a critical parameter that can be influenced by external factors such as xenobiotics, which affect processes such as photosynthesis and nutrient uptake. For example, the C:N ratio in *Scenedesmus obliquus* was found to significantly increase when exposed to 0.05 mg/L sulfonamides, but this ratio decreased at concentrations higher than 0.15 mg/L [38].

MCs, secondary metabolites produced by microalgae such as *M. aeruginosa*, are composed primarily of C and N. The intracellular C:N ratio is critical in regulating the synthesis and composition of these toxins [39]. The availability of C influences photosynthesis through carbon concentrating mechanisms, glycogen storage, and altered ratios of photosystems I and II [40]. Moreover, the balance between C and N within the cell directly impacts the activity of enzymes such as glutamine synthetase and glutamate synthase. These enzymes are involved in pathways closely linked to MC production, particularly through their regulation of 2-oxoglutarate and ammonium levels, both of which are crucial modulators of MC synthesis in *M. aeruginosa* [40]. The C:N ratios of the three MC congeners, MC-LR, MC-RR, and MC-YR, are 4.90, 3.77, and 5.20, respectively. Changes in intracellular C:N ratios could alter the synthesis and composition of these MC congeners. For example, an increase in C in *M. aeruginosa* may enhance the participation of carbon in the Calvin cycle or glycolysis, potentially increasing the availability of C skeletons for MC synthesis [19]. Conversely, a decrease in intracellular N could lead to a shift in the synthesis toward nitrogen-poor congeners like MC-YR, as the cell reallocates its carbon resources.

### 3.3. Effects of CIP on Amino Acid Content

**Figure 3:**
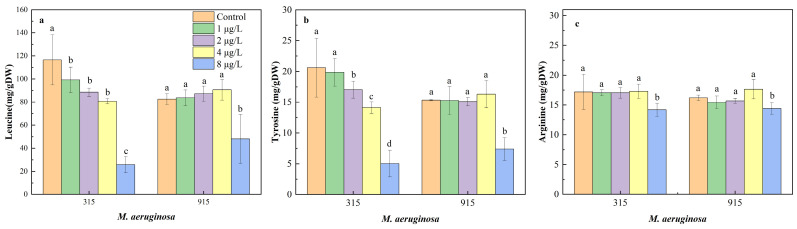
Effects of CIP concentrations on leucine (**a**), tyrosine (**b**), and arginine (**c**) content in FACHB-315 and FACHB-915 after 18 days of cultivation. Different letters indicate significant differences (*p* < 0.05) between the control and experimental groups.

The contents of leucine, arginine, and tyrosine in *M. aeruginosa* exposed to different concentrations of CIP are shown in Figure 3. The effects of CIP on leucine (Figure 3a) and tyrosine (Figure 3b) content exhibited similar trends. Specifically, the leucine content in FACHB-315 decreased by 14.86%, 23.95%, 30.67%, and 77.86% when exposed to CIP concentrations at 1, 2, 4, and 8 μg/L, respectively, while the content of tyrosine decreased by 3.64%, 17.31%, 31.50%, and 75.59% at 1, 2, 4, and 8 μg/L, respectively. However, the content of leucine and tyrosine in FACHB-915 significantly decreased only at 8 μg/L CIP by 41.79% and 51.86%, respectively, compared with the control. As for arginine (Figure 3c), no significant decreases were observed in either FACHB-315 or FACHB-915 at CIP concentrations lower than 4 μg/L. But the arginine content at 8 μg/L CIP decreased by 17.54% and 11.20% in FACHB-315 and FACHB-915, respectively.

**Figure 4:**
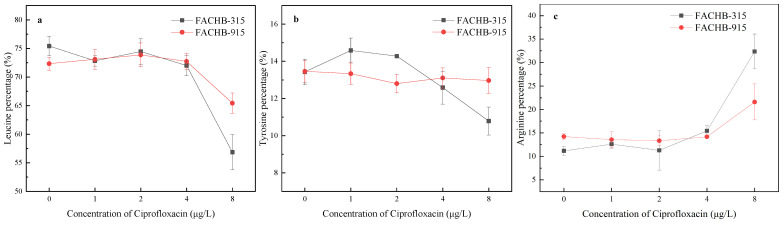
Effects of CIP concentrations on the composition of leucine (**a**), tyrosine (**b**), and arginine (**c**) in FACHB-315 and FACHB-915 after 18 days of cultivation.

As shown in Figure 4a, the percentage of leucine in both FACHB-315 and FACHB-915 decreased with increasing CIP concentrations. The percentage of leucine in FACHB-315 and FACHB-915 decreased to 56.86% and 65.43% at 8 μg/L CIP, respectively. In contrast, the percentage of tyrosine notably increased at the same concentrations to 32.35% and 21.61%, respectively.

MCs are cyclic heptapeptides typically composed of seven amino acids, with the most variability occurring at positions 2 and 4, where different L-amino acids can be substituted [41]. The structural diversity of MCs in *M. aeruginosa* is mainly attributed to the constitution of amino acids, such as leucine (L), tyrosine (Y), and arginine (R), among others. When these amino acids are integrated into the variable positions of the cyclic peptide structure, they result in different MC congeners, including MC-LR, MC-YR, and MC-RR, among others. Therefore, changes in amino acid content may lead to variations in MC production.

The synthesis of amino acid in algae can be influenced by both xenobiotics and environmental conditions. For instance, in *Raphidocelis subcapitata*, leucine synthesis was found to be downregulated upon exposure to erythromycin, while arginine synthesis was upregulated [42]. Similarly, exposure to polystyrene nanoplastics was found to enhance tyrosine synthesis in *Phaeodactylum tricornutum* [43]. The contents of leucine and tyrosine in *M. aeruginosa* under CIP exposure were observed to significantly decrease, while arginine levels remained relatively stable. These changes led to a notable decrease in the percentage of leucine and a relative increase in arginine proportion.

These findings suggest that CIP primarily affects leucine synthesis, and the reduction in leucine could potentially lead to decreased production of MC-LR, respectively. This implies that CIP exposure not only alters the synthesis of specific amino acids but may also affect the overall composition and concentration of MCs within *M. aeruginosa*, which could influence the toxicity profile of cyanobacterial blooms in natural aquatic systems.

### 3.4. Effects of CIP on mcyB Genes Expression

**Figure 5:**
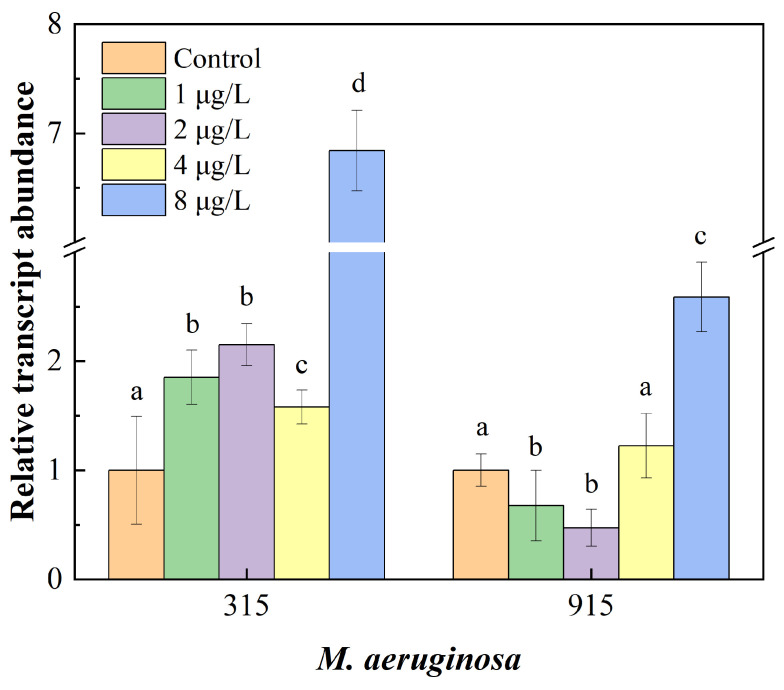
Effects of CIP concentrations on the *mcyB* genes expression in FACHB-315 and FACHB-915 after 18 days of cultivation. Different letters indicate significant differences (*p* < 0.05) between the control and experimental groups.

The effects of CIP concentrations on the *mcyB* gene expression in *M. aeruginosa* are presented in Figure 5. The expression levels of the *mcyB* gene in FACHB-315 significantly increased under CIP exposure. At concentrations of 1, 2, 4, and 8 μg/L, the expression levels were 1.85, 2.15, 1.58, and 5.57 times higher than the control group, respectively. For FACHB-915, the expression levels decreased under lower CIP concentrations but increased significantly at 8 μg /L, reaching 2.59 times higher than that of the control group.

The *mcyB* gene is a part of the *mcy* gene cluster, which is responsible for the synthesis of MCs in *Microcystis* species. Previous studies have shown that MCs are not produced if the *mcy* gene is absent or inactive [44]. The expression of the *mcy* gene cluster, and consequently the production of MCs, may be influenced by various environmental factors and xenobiotics, including antibiotics [29], heavy metals [45], as well as temperature and nutrients [46]. The response of *mcyB* gene expression to CIP exposure observed in this study was consistent with previous research. For example, *mcyB* expression was significantly stimulated by amoxicillin in *M. aeruginosa*, leading to enhanced MC production [47]. The upregulation of *mcyB* in response to CIP in *M. aeruginosa* indicated that MC synthesis was regulated under CIP exposure on the genetic level. Moreover, the upregulation of *mcyB* suggests that CIP may not only affect the growth of *M. aeruginosa* but also modulate its toxicity by increasing MC production. This has serious ecological and public health implications, particularly in eutrophic water bodies where *Microcystis* blooms are prevalent.

### 3.5. Effects of CIP on Cellular MCs

**Figure 6:**
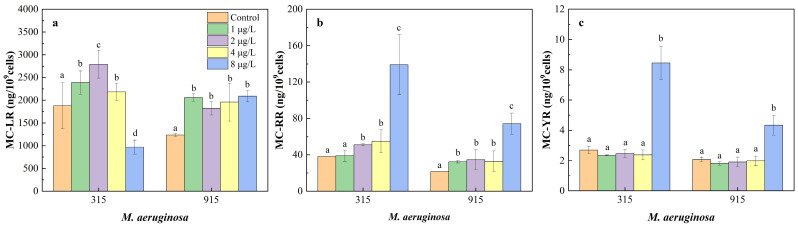
Effects of CIP concentrations on the content of cellular microcystin in *M. aeruginosa* after 18 days of cultivation ((**a**): MC-LR, (**b**): MC-RR, (**c**): MC-YR). Different letters indicate significant differences (*p* < 0.05) between the control and experimental groups.

The contents of MC-LR, MC-RR, and MC-YR in *M. aeruginosa* exposed to different concentrations of CIP were determined (Figure 6a–c). The MC-LR content in both FACHB-315 and FACHB-915 significantly increased at CIP concentrations below 4 μg/L. However, the MC-LR content in FACHB-315 significantly decreased at 8 μg/L CIP, while it continued to increase in FACHB-915 at that concentration. Regarding MC-RR and MC-YR, their contents showed a significant increase at high CIP concentrations. The highest increase in MC-RR content was 264.08% for FACHB-315 and 244.90% for FACHB-915 at 8 μg/L CIP. Similarly, MC-YR contents increased by 213.88% in FACHB-315, at the same concentration.

**Figure 7:**
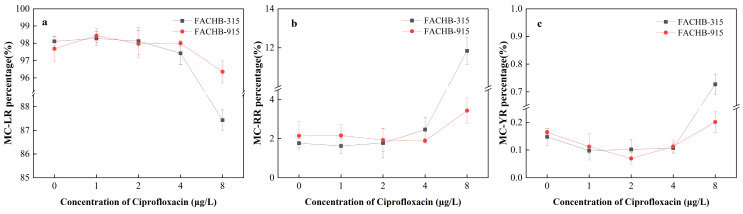
Effects of CIP concentrations on the composition of cellular microcystin in *M. aeruginosa* after 18 days of cultivation ((**a**): MC-LR, (**b**): MC-RR, (**c**): MC-YR).

Figure 7a–c illustrates the effects of CIP on the composition of MC-LR, MC-RR, and MC-YR in *M. aeruginosa*. MC-LR is the predominant MC congener in *M. aeruginosa.* The percentage compositions of MC-LR, MC-RR, and MC-YR in FACHB-315 were 98.10%, 1.76%, and 0.14%, respectively, without CIP exposure, and were 97.69%, 2.15%, and 0.16% in FACHB-915, respectively. At CIP concentrations below 4 μg/L, no significant changes in the percentage composition of these three MCs were detected. However, at 8 μg/L CIP exposure, the percentage of MC-LR significantly decreased to 87.43% in FACHB-315, while the percentages of MC-RR and MC-YR increased to 11.84% and 0.73%, respectively. As to FACHB-915, the percentage of MC-LR significantly decreased to 96.36%, while the percentages of MC-RR and MC-YR increased to 3.44% and 0.20%, respectively.

MCs have a half-life of 90–120 days in natural water, posing a significant threat to human health through various exposure pathways [48]. Exposure to CIP can induce the accumulation of reactive oxygen species (ROS) within cells, which in turn can stimulate MC synthesis in toxic cyanobacterial strains as a protective mechanism against oxidative stress [21]. Under environmental stress, the expression of the *mcy* gene cluster and the synthesis of related proteins can be enhanced, promoting MC production [4]. The upregulation of protein export membrane proteins and outer membrane efflux proteins, as well as increased cell permeability or cell breakdown under antibiotic stress, may facilitate MC release in *M. aeruginosa* [49,50]. However, the growth of *M. aeruginosa* is significantly inhibited at high CIP concentrations (8 μg/L), reducing the release of MCs. In addition, most previous research mainly focused on the effects of xenobiotics on the release of MC-LR or total MCs by *M. aeruginosa* [51,52]. For example, moxifloxacin and gatifloxacin significantly increased the release of total MCs in *M. aeruginosa* at 10 μg/L [4], and erythromycin and sulfamethoxazole were found to enhance the production and release of MC-LR [32]. Additionally, antibiotic mixtures were shown to significantly increase MC production in *M. aeruginosa* when exposed to concentrations ranging from 50 to 500 ng/L [53]. However, limited studies have investigated the impact of antibiotics on the production of different MC congeners. In the few available studies, three tetracyclines were reported to affect cellular MC congeners. For example, tetracycline hydrochloride increased cellular MC-LR concentrations while reducing MC-RR in *M. aeruginosa*. Chlortetracycline hydrochloride simultaneously decreased cellular MC-LR and MC-RR concentrations. In contrast, oxytetracycline hydrochloride increased MC-LR concentrations without affecting MC-RR levels [22]. These findings highlight the necessity of research related to the impact of antibiotics on the production of different MC congeners.

The toxicity of different MC congeners is closely related to their structure. For example, the LD_50_ of MC-RR for mice is 600 μg/L, whereas for MC-LR, it is only 50 μg/L [19]. To date, more than 300 MC congeners have been identified, with many additional congeners still remaining to be discovered [54]. MC-LR is the primary MC variant produced by both FACHB-315 and FACHB-915, among the three most studied variants (MC-LR, MC-RR, and MC-YR) [25]. The concentration and composition of different MC congeners in *M. aeruginosa* were changed by CIP exposure. In FACHB-315, the production of MC-LR was stimulated at CIP concentrations lower than 4 μg/L, but inhibited at 8 μg/L, while the production of MC-RR and MC-YR was stimulated at 8 μg/L. However, the production of MC-LR, MC-RR, and MC-YR was stimulated in FACHB-915 across all treatment groups. As a consequence, the composition of MC-LR, MC-RR, and MC-YR did not significantly change at CIP concentrations below 4 μg/L, but at 8 μg/L, the percentage of MC-LR decreased significantly, while the percentages of MC-RR and MC-YR increased in both FACHB-315 and FACHB-915.

Previous studies have shown that the excess C in algal cells can be directed into the Calvin cycle or glycolysis, leading to an increase in the C skeletons for MC synthesis [20]. When the C:N ratio increases, the relative N content in cells decreases, and these C skeletons in algal cells are more likely to be used to synthesize N-poor MCs, such as MC-YR, which has a C:N ratio of 5.2 [20]. However, in this research, the compositions of both MC-RR and MC-YR were increased under high concentrations of CIP exposure in *M. aeruginosa*. The C:N ratio of MC-RR was 3.77, indicating that nitrogen-rich MC-RR was synthesized when the C:N ratio increased. The synthesis of MCs is not only influenced by C skeletons but also affected by amino acid synthesis. The syntheses of leucine, tyrosine, and arginine were all inhibited under CIP exposure, but the composition of these amino acids was changed. The increase in arginine percentage in algal cells led to the promotion of MC-RR synthesis, while the decrease in leucine percentage reduced the production of MC-LR. This indicates that both the ratios of C:N and amino acids composition play crucial roles in determining the concentration and composition of MCs. Despite the changes in the proportions of MC congeners released by *M. aeruginosa* during this study, MC-LR remained the dominant congener. But many other MC congeners have been reported as the primary MC congeners in some lakes [17,18]. Therefore, assessing toxicity risk based solely on the total MC concentration or MC-LR levels has significant limitations. To accurately determine the actual toxicity and pollution levels in aquatic environments, it is essential to understand the distribution of different MC congeners in the water.

## 4. Conclusions

This study investigated the effects of CIP on growth, carbon and nitrogen balance, amino acid composition, gene expression, and microcystin production in two toxin-producing strains of *Microcystis aeruginosa* (FACHB-315 and FACHB-915). The findings demonstrated that CIP significantly influenced the physiological and biochemical processes of *M. aeruginosa*. Specifically, this study revealed that CIP exposure inhibited the growth of both strains, with FACHB-315 being more sensitive than FACHB-915. The intracellular C:N ratio increased in response to CIP, particularly in FACHB-315, altering the synthesis and composition of amino acids and microcystins (MCs). Leucine and tyrosine content decreased significantly under CIP exposure, while arginine levels remained relatively stable, suggesting that CIP affected the synthesis pathways of specific amino acids, leading to a reduction in MC-LR levels and an increase in MC-RR and MC-YR production. The upregulation of the *mcyB* gene under CIP exposure indicated that antibiotic stress could enhance MC synthesis at the genetic level, contributing to the increased toxicity of cyanobacteria. The results emphasize the importance of understanding how antibiotics regulate microcystin synthesis and composition in harmful cyanobacteria, which has significant implications for environmental management and human health in the context of cyanobacterial blooms. Further research is needed to explore the long-term impact of antibiotic exposure on microcystin production and its ecological impacts.

## Data Availability

The original data presented in the study are included in the article. Further inquiries can be directed to the corresponding author.
